# The implementation of take-home naloxone: Lessons learned from a 3-year take-home naloxone project in Germany

**DOI:** 10.1186/s12954-025-01281-1

**Published:** 2025-07-18

**Authors:** Simon Fleißner, Larissa Steimle, Dirk Schäffer, Bernd Werse, Daniel Deimel, Maria Kuban, Heino Stöver

**Affiliations:** 1 Faculty of Social Sciences, Technical University Nuernberg Georg Simon Ohm, Keßlerplatz 12, 90489 Nuremberg, Germany; 2 Institute of Addiction Research, Frankfurt University of Applied Sciences, Nibelungenplatz 1, 60318 Frankfurt a.M, Germany; 3 German AIDS Federation, Wilhelmstr. 138, 10963 Berlin, Germany; 4 Department of Psychiatry and Psychotherapy, Medical Faculty, LVR, University Hospital Essen, University of Duisburg-Essen, Virchowstraße 174, 45147 Essen, Germany

**Keywords:** Opioid overdose, Overdose emergency training, Harm reduction, Take-home Naloxone, Implementation

## Abstract

**Background:**

Take-home naloxone (THN) can prevent deaths related to opioid overdoses. Despite the first THN project in Germany in 1998, the availability of naloxone for people who use opioids (PWUO) is still scarce. We present the results of the German-wide THN-project NALtrain, which aimed to implement THN nationwide. Firstly, we present data collected during NALtrain and secondly, we use this data to critically reflect on the project and thereby draw conclusions that could inform future THN projects.

**Method:**

NALtrain was conducted between July 2021 and June 2024. Descriptive statistical analysis of the documentation of 74 train-the-trainer events and following naloxone trainings conducted by the trained staff were carried out.

**Results:**

864 staff members from approximately 373 organizations (mainly harm reduction services) participated in 74 train-the-trainer courses. Of the 373 organizations 123 conducted 784 naloxone trainings for PWUO and reached 2,333 PWUO, of whom 1,451 received THN. The goal of training 800 staff members was met, while the goals of reaching 400 organizations and 10,000 PWUO were missed. The implementation of THN is unevenly distributed across the German federal states, especially concentrated in Bavaria. The core learnings are that the prescription-only status of THN leads to extra organizational efforts and hinders the availability of THN for individuals with the highest risk of overdose.

**Conclusion:**

Considering the proportion of organizations offering THN, they can still be classified as “early adopters”. These may serve as role models for the broader majority. Free available THN and centrally coordinated support of implementation including recurring follow-up can be key to a broader availability of THN in Germany. In future initiatives physicians and medical settings should be prioritized.

## Highlights

864 staff members from approximately 373 organizations were trained.

About 123 organizations conducted 784 naloxone trainings and reached 2,333 people who use opioids (PWUO), of whom 1,451 received THN.

Prescription-only status of THN hinders availability of THN for PWUO.

Physicians and medical settings should be involved in an early stage and prioritized in future THN projects.

Centrally and ongoing coordination, support and follow-up is vital for long term implementation of THN.

## Background

In 2023, the number of drug-related deaths in Germany reached a new peak, with 2,227 cases. Similar to previous years, the majority (56%) of these deaths were associated with opioid use [1]. Across Europe, drug-related deaths are also increasing, with the European Drug Agency (EUDA) estimating that 76% of these involve opioids [2]. Naloxone reverses the effects of opioids if administered timely, and can therefore prevent opioid-related deaths [3–11]. Take-home naloxone (THN) refers to a public health intervention in which naloxone is provided to laypersons at risk of witnessing an overdose, along with education on how to recognize and respond to opioid overdoses. Model calculations confirm reduced mortality due to THN [6, 12–14] and studies show that naloxone is effectively administered by laypersons [5, 8, 15–17]. Laypersons can identify overdoses, administer naloxone correctly and use recommended first-aid techniques in case of a drug-emergency [17, 18]. Adverse events are rare and can be mitigated by adequate training [9, 19]. Furthermore, THN does not lead to more or more risky drug consumption of the reached people who use opioids (PWUO) [20, 21]. Even though it has been proven that THN is a critical public health intervention to the opioid epidemic, it still is rarely implemented in many European countries [22, 23].

It is estimated that 166,000 individuals with opioid addiction are currently living in Germany, varying widely between the 16 federal states. About 50% of these are in opioid-agonist-therapy (OAT) [24]. The number of PWUO without opioid addiction in Germany is unknown. Further, there is no exact number of facilities that would be suitable to reach PWUO with THN. The German Drug Service Registry estimates 325 residential rehabilitation facilities and 1,230 outpatient facilities in Germany, including about 300 low-threshold facilities [25]. Additionally, there are 402 psychiatric departments, 209 specialized psychiatric hospitals or hospitals with corresponding departments, and 2,436 physicians offering opioid-agonist-treatment (OAT) [26]. There are also 172 correctional facilities [27]. However, it is challenging to estimate how many of these facilities would be suitable to reach PWUO with THN, because not all of these organizations work with PWUO, and further differentiation is not possible based on available data. Since 2018, naloxone has been available as nasal spray on prescription only. The naloxone-nasal spray can only be prescribed to individuals with opioid addiction and is covered by health insurance. Prescription on the cost of health insurance is only allowed for physicians licensed under the statutory health insurance system. Therefore, prescriptions by physicians of drug services are mostly not covered by insurance, and naloxone must be paid for either by the PWUO or by the drug services. Naloxone is available in pharmacies only.

One of the world’s first THN programs was launched in Berlin in 1998 [28], but THN access is still low in Germany. Since 2014, smaller local projects led by harm reduction services have been conducted in several cities. It remains unknown how long and to what extent THN was provided in these harm reduction services. In 2017, a THN program was initiated in the federal state of Saarland aiming to reach 50 PWUO annually and is still operating. Between 2018 and 2020 a pilot project was conducted in the federal state of Bavaria (BayTHN) reaching 537 PWUO [17, 29]. The implementation of THN in Germany is patchy and there is almost no long-term funding. From July 2021 to June 2024, the federal pilot project NALtrain (“design, implementation, and evaluation of a scientific model project for conducting nationwide quality-assured take-home naloxone trainings: national early warning system”) was carried out. The project’s organizers of NALtrain were Akzept e.V. (national umbrella organization for harm reduction), the German AIDS Federation, and the Institute of Addiction Research Frankfurt. Studies show that the train-the-trainer model can be sufficient for scaling up the knowledge and intent for conducting overdose prevention trainings with naloxone in staff members of low-threshold facilities [15, 30]. The German AIDS Federation was already experienced in offering train-the-trainer events in the field of harm reduction on the topics of “safer use” or “drug emergency”. This approach was utilized by NALtrain to scale up THN in Germany. NALtrain aimed to expand the distribution of THN and enable drug- and AIDS-service organizations across the country to offer naloxone training with the goal to train 800 staff members from 400 organizations to reach 10,000 PWUO with training and THN. To evaluate the implementation of the NALtrain project, the number of organizations and staff involved in training at various locations and the number of naloxone trainings and naloxone uptake among PWUO were examined. Following Skivington et al. [31 p. 5] in the “new framework for developing and evaluating complex interventions” we try to present “[…] equivocal findings that nevertheless inform important decisions where evidence is sparse”. Therefore, the aim of this article is twofold: Firstly, we present data collected during NALtrain and secondly, we use this data to critically reflect on the project and thereby draw conclusions that could inform future THN projects.

## Method

### Design and setting

Naltrain was a German-wide implementation project consisting of two parallel components conducted between July 2021 and June 2024. The first component involved manualized train-the-trainer events (hereafter referred to as “training”), where staff from drug and AIDS service organizations were trained to conduct overdose emergency training including distribution of naloxone to PWUO (hereafter referred to as “naloxone trainings”). The trainings duration was between four and five hours and included the following topics: opioids, overdose, naloxone, first-aid measures, reaching PWUO, didactic and organizational issues. The naloxone training for PWUO included the following topics: naloxone, overdose, first-aid measures and emergency calls. The naloxone training could be done in group settings or with single PWUO. Due to the above-described regulations the local drug services were needed to organize the distribution of naloxone to the participating PWUO. No funding to the participating organizations through NALtrain was available. Naloxone was covered by health-insurance or the harm reduction services. On average, each staff member would need to train 12–13 PWUO, or each organization 25 PWUO to reach the goal of 10,000.

### Measures

The trainings for staff members were documented by paper and pencil with lists of participation. The documentation included: name of organization and area of work. The naloxone trainings for PWUO were documented by the drug services either with paper and pencil or online (SoSciSurvey). The documentation included: number of participants, distributed naloxone, name of organization, city where the training took place and date.

### Inclusion criteria

All organizations associated with the German AIDS Federation and Akzept e.V. have been contacted via e-mail. Within the network of these two organizations, there are mainly low-threshold harm reduction services. This results in targeting PWUO with higher risk of overdose like people who inject drugs or being part of an open drug scene. Furthermore, eligible newsletters were approached to inform about NALtrain and possibilities to participate. All organizations who work in the area of opioid use were eligible to participate, even if they already offered THN. All PWUO could participate in the naloxone trainings carried out by the harm reduction services. Further inclusion criteria were individually set by the harm reduction services (e.g. intoxication).

### Statistical analysis

Data was collected between December 2021 and June 2024. Data was analyzed descriptively using SPSS (29.0.1.0) and Excel (Microsoft 365). The two data collection processes could not be linked. The documentation of the second component was conducted externally by the Centre for Drug Research in Frankfurt. The overall project results were published as a project report [32].

### Ethical considerations

This study did not require ethical approval, as it was conducted using only anonymized data. Specifically, anonymized participant lists from train-the-trainer events and records of naloxone trainings were used. No personally identifiable information was processed.

## Results

### Trainings

A total of 74 manualized training courses were offered in 38 different cities, training 864 staff members to provide naloxone training. Training sessions had a minimum of 2 participants and a maximum of 23, with an average of 11.7 participants per session (SD = 4.9). At the organizational level, estimates were required due to imprecise naming and distinctions between organizations. Staff from approximately 373 different organizations participated, with at least one person trained from each organization and a maximum of 21 participants from a single organization. On average, 2.3 staff members per organization were trained (SD = 2.5). Table 1 shows the number of staff trained per 1,000 PWUO by federal states.

**Table 1:** Trained staff by federal states for 1,000 PWUO

Federal State	Staff	Estimate PWUO [24]	Staff per 1,000 PWUO
Hamburg	17	8,847	1.9
Lower Saxony	36	16,794	2.1
Saxony-Anhalt	4	1,467	2.7
Bremen	11	3,745	2.9
Hesse	52	16,042	3.2
Mecklenburg-Western Pomerania	2	538	3.7
North Rhine-Westphalia	233	53,851	4.3
Schleswig-Holstein	35	6,961	5.0
Baden-Württemberg	123	21,832	5.6
Brandenburg	2	248	8.1
Rhineland-Palatinate	38	4,672	8.1
Bavaria	137	16,713	8.2
Saxony	14	1,342	10.4
Berlin	133	10,943	12.2
Thuringia	27	819	33.0

**Fig. 1:**
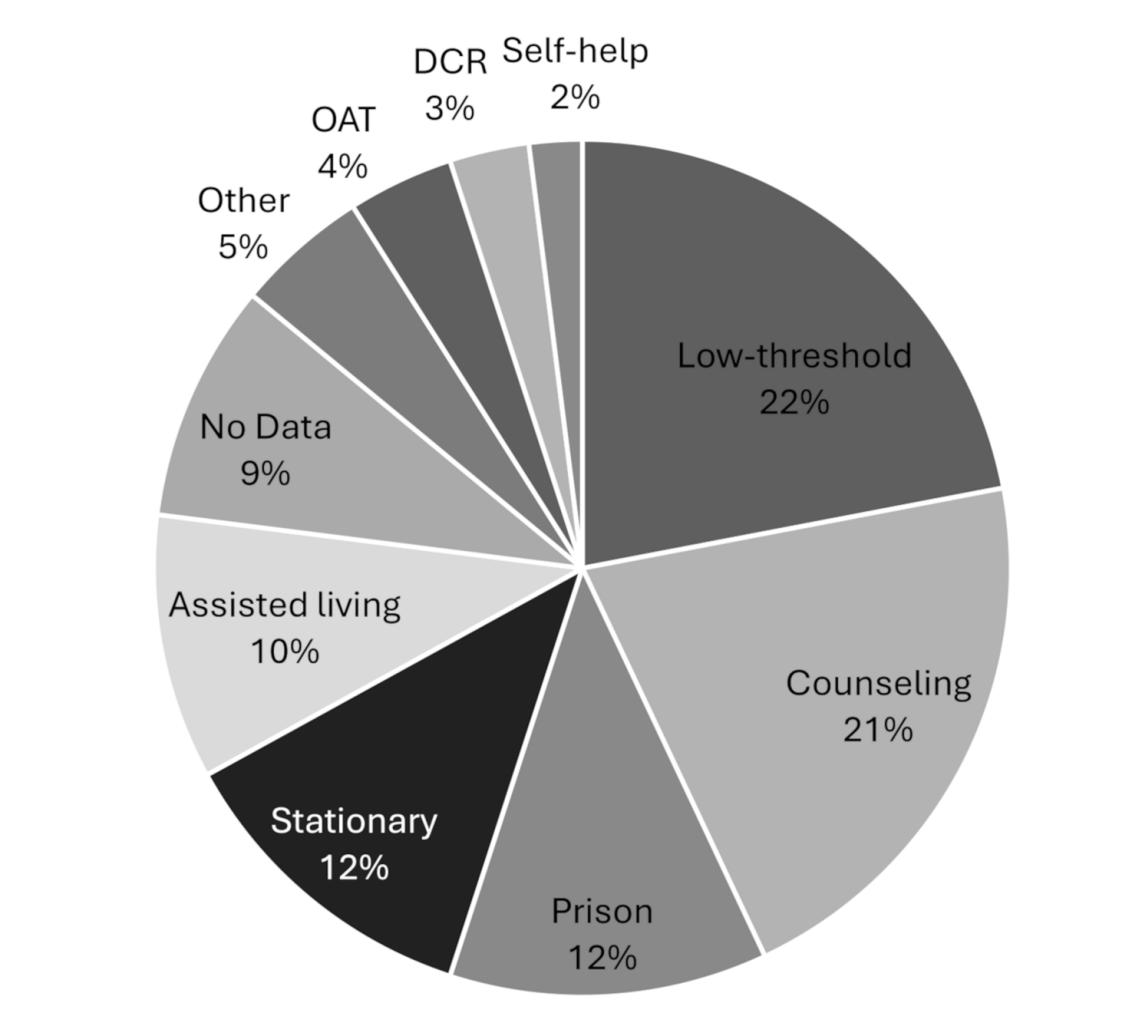
Work areas of trained staff (*n* = 864)

Participants predominantly worked in low-threshold facilities *n* = 191 (22%) and counseling services *n* = 180 (21%). Additionally, *n* = 104 (12%) worked in prison and stationary treatment each, and *n* = 87 (10%) worked in assisted living facilities. With *n* = 34 (4%) fewer participants were employed in medical settings (primarily opioid-agonist-therapy (OAT)) or *n* = 26 (3%) in supervised drug consumption rooms (DCR) and *n* = 17 (2%) came from self-help groups (see Fig. 1).

### Naloxone trainings

A total of 2,333 PWUO were trained, of whom 1,451 received naloxone following the naloxone training. On average, 2.7 PWUO were reached per trained staff member. Participating organizations documented 784 naloxone trainings, conducted by approximately 123 different drug services across 79 cities in Germany. On average, each organization conducted 6.4 naloxone trainings (SD = 10.5) and reached 19 PWUO (SD = 27.8). The minimum number of naloxone trainings and PWUO reached per facility was 1. The maximum number of naloxone trainings conducted by a single organization was 63, while the highest number of PWUO reached was 150.

The naloxone trainings were predominantly concentrated in three federal states, with Bavaria accounting for the largest share. The proportion of PWUO who received naloxone varied by state. Bavaria had the highest rate of naloxone distribution, with *n* = 825 (73.6%) of PWUO receiving naloxone trainings. The highest access to THN in relation to 1,000 PWUO are also in Bavaria (see Table 2). In the states of Saarland and Brandenburg, no naloxone trainings were documented with NALtrain.

**Table 2:** Naloxone trainings by federal states

Federal State	Trainings	Participants	Avg. Participants/Training	Naloxone Recipients	Participants with Naloxone	THN per 1,000 PWUO
Bavaria	482	1,126	2.3	825	73.3%	49.4
Baden-Württemberg	147	524	3.6	322	61.5%	14.7
North Rhine-Westphalia	85	365	4.3	203	55.6%	3.8
Berlin	20	135	6.2	24	17.8%	2.2
Hamburg	15	42	2.8	11	26.2%	1.2
Hesse	10	37	3.7	28	75.7%	1.7
Lower Saxony	10	33	3.3	6	18.2%	0.4
Rhineland-Palatinate	4	16	4.0	14	87.5%	3.0
Schleswig-Holstein	3	26	8.7	1	3.8%	0.1
Bremen	3	10	3.3	8	80.0%	2.1
Saxony	2	7	3.5	3	42.9%	2.2
Mecklenburg-Western Pomerania	2	6	3	6	100%	11.2
Thuringia	1	6	6	0	0%	0.0

**Fig. 2:**
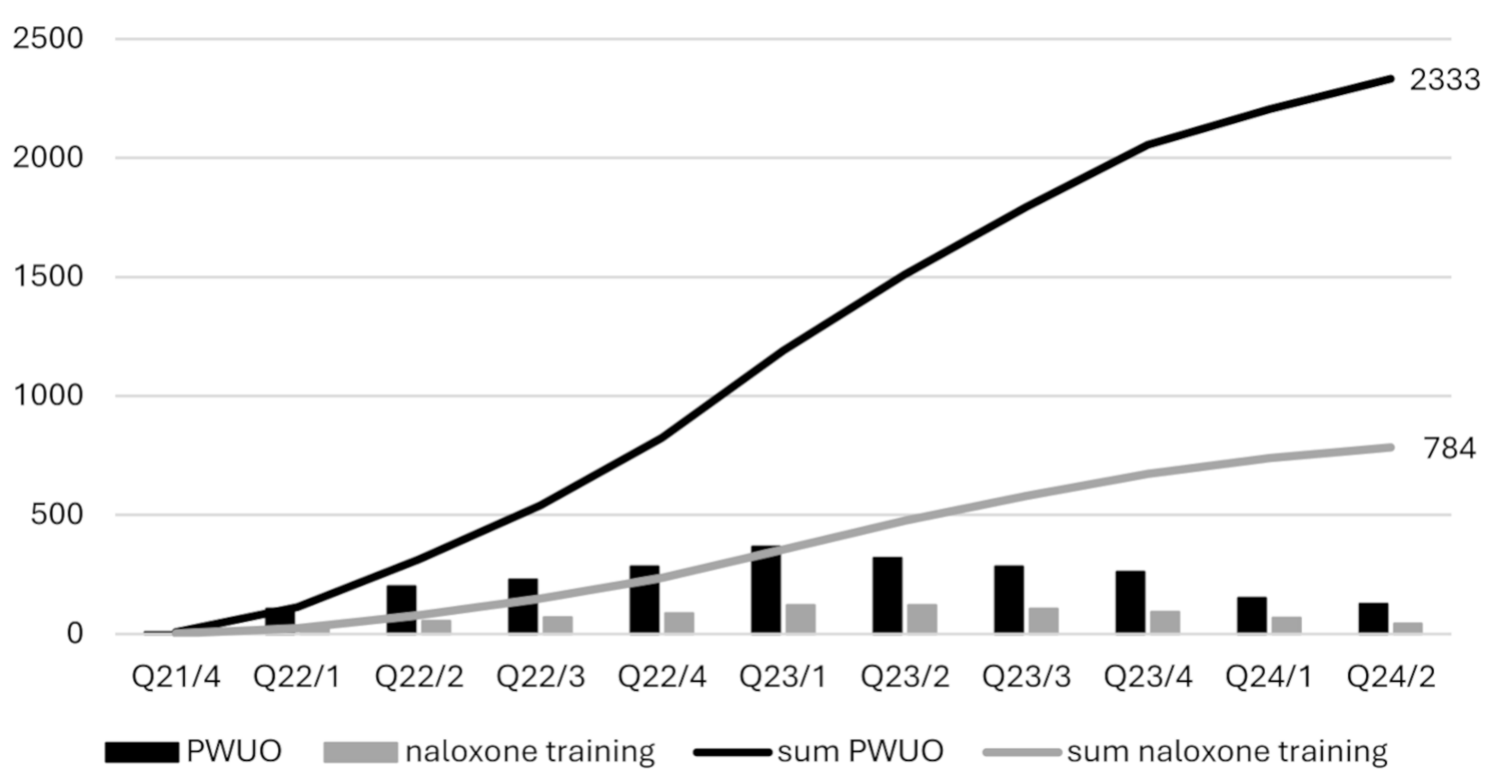
Number of naloxone trainings and reached PWUO by quarters

During the first six quarters, until the first quarter of 2023 (Q23/1), the number of naloxone trainings and trained PWUO increased steadily. However, from Q23/2 onwards, these numbers began to decline (See Fig. 2).

## Discussion

### Organizations reached

NALtrain’s goals were met with training 864 staff members and almost met with involving approximately 373 organizations. With roughly 373 organizations involved in NALtrain, only a fraction of eligible facilities in Germany was reached. Low-threshold facilities and correctional institutions were well represented, as reflected in the participants’ reported areas of work. Medical facilities, particularly OAT physicians and stationary treatment facilities, were less engaged. The strength of the NALtrain project consortium network is reaching the harm reduction services, including low-threshold and counseling services. Harm reduction services, like facilities that provide needle and syringe exchange or drug consumption rooms, can play a vital role in distributing naloxone [33, 34]. Targeting these with train-the-trainer programs means reaching people at risk of overdose. Beside the low-threshold setting, the example of the national THN-program in Scotland shows the important role prisons have in scaling-up THN [4]. BayTHN has already demonstrated the feasibility of naloxone distribution in the setting of correctional facilities in Germany [35]. NALtrain has engaged prisons, but throughout the project the federal ministries of justice responsible for prison healthcare were mostly hesitant to distribute naloxone on release, even after prison staff members participated in a training course. Without immediate naloxone distribution THN in prisons will fail to address the first days after release with highest risk of overdose [36]. Abstinence-based treatment facilities were originally not targeted by the networks of NALtrain. It is therefore particularly noteworthy that some still participated and were able to successfully offer THN. Van and colleagues have reported that this setting can reach people at risk of opioid overdose [37]. However, it is likely that only a small fraction of the relevant abstinence-based treatment facilities was involved in NALtrain, and these could become the focus of future train-the-trainer projects. Such a project may need a tailored training manual for the specific inpatient setting. Due to the need of physicians for the prescription of naloxone, the OAT is crucial and can play an important part for scaling-up THN in Germany. Beside that an OAT itself lowers the risk of overdose [38] it can also reach people at risk [18, 39]. The number of OAT physicians in Germany is declining. As a result the implementation of THN in OAT is challenging [40]. NALtrain failed to engage the setting of OAT, which contributed to not reaching 10,000 PWUO. Setting THN as a standard in treating people with opioid addiction could help establish THN in medical settings in Germany [11]. This could also support drug services to establish cooperation for the prescription of naloxone. Examining perceptions about THN within medical settings would help to understand the barriers for wider implementation. This could also be relevant for harm reduction services, because the uptake and prioritization of THN still seems to be low in most of these organizations. Differences were also observed in the distribution of trained staff across federal states in relation to the number of PWUO. While reaching PWUO in urban areas with harm reduction services is feasible, in more rural regions and federal states a different approach could be utilized. Dropping of naloxone by emergency departments or ambulance after an opioid overdose could reach PWUO not only, but also in rural areas [41, 42]. Both settings were not targeted by any THN-project in Germany yet. To understand the availability of THN in the population of people at risk of overdose, studies of open drug scenes could include questions about THN [43]. This information can inform future THN efforts and result in local tailored THN-strategies.

### PWUO reached

With an average of 6.2 trained PWUO per participating institution and 2.7 per trained staff member, the targeted 25 per institution and 12–13 per staff member were not achieved. In consequence, the goal of reaching 10,000 PWUO with THN was largely missed. The documented naloxone trainings were conducted by only about 123 different institutions. This suggests that approximately 250 institutions have trained staff but have not yet offered naloxone training for PWUO or evaluated these. It is not possible to link the two datasets, missing out valuable information on who of the trained staff members conducted naloxone trainings for PWUO and to what extent. The number of naloxone distributed per 1,000 PWUO in the federal states shows that the naloxone distributed in Bavaria is by far the highest of all federal states. Two reasons might have significantly contributed to the better access in Bavaria. First, Bavaria is the only federal state with sufficient funding for THN. Lack of funding is one major barrier for harm reduction measures in general [44]. The HEALing Communities Study (HCS) has also shown that sufficient funding can be key to an increase in THN availability [45]. The NALtrain data shows that in 22 of 79 cities where naloxone trainings were documented, there was financial support from the federal state (e.g., Bavaria and Rhineland-Palatinate) or the municipality (e.g., Mannheim). These 22 cities account for 50% of the reached PWUO. Second, with BayTHN there was a preceding project, and the five organizations already participated in BayTHN reached 534 PWUO during the study period of NALtrain. NALtrain can be understood as a follow-up for these five institutions and emphasizes the importance of central and long-term guidance of THN. The examples of Norway and the HCS in the US underline the need and effectiveness of centrally governed support [45, 46]. A sustained implementation of naloxone training would potentially lead to an exponential or at least linear increase in the number of reached PWUO each quarter (see Fig. 2). This can for example be seen in the data of the national THN-program in Scotland [4] or a large-scale THN-program in Ohio, US [47]. However, such a trend was only observed until Q23/1. After this point, the number of reached PWUO declined. This suggests that institutions are discontinuing their naloxone training programs or training fewer PWUO than at the beginning, already during the studied period. NALtrain was probably not able to offer enough follow-up support. Only material for information (e.g. Flyer, Poster) was available and a person responsible for questions. Even if not explicitly documented in the presented data, throughout the project, finding physicians to prescribe naloxone was a major issue.

### Lessons learned

In a narrative review, Dee et al. [44] found that “stigma, lack of education and knowledge, and lack of access to resources for long-term management” [44] are major barriers for the implementation of harm reduction methods in general and therefore also for THN in Germany. Overcoming stigma is, if at all, only partially mitigated by a THN project. Education and knowledge were the core concept of NALtrain by offering training for the staff. Allocating resources to where it is needed most can be key, as resources are always limited. Doe-Simkins and Wheeler [48] state three evidence-based practices of naloxone distribution: (1) directly to PWUO via low-threshold, namely syringe service programs, (2) THN on release from prison and (3) coprescribing naloxone with opioids for people at high risk. Similar to Norway, a central (governmental) body could oversee and support the implementation process [49] and evaluate and adapt the implementation. As coprescribing naloxone could be rarely achieved within NALtrain, targeting the medical setting and educating physicians should be a central task of future initiatives.

The prescription-only status of THN accounts for an increase in organizational workload, because of the obligatory involvement of a physician. Due to the prescription only status, the trialability is lower, it is more complex and the operating costs of THN are therefore higher. Furthermore, the efforts needed to involve physicians and their willingness to prescribe naloxone were underestimated at the beginning of NALtrain and is evident by only 4% of trained staff in the context of OAT. The lack of randomized controlled trials and meta-analysis of the effectiveness of THN to reduce the mortality of PWUO may be one reason for caution of physicians to be involved in THN. Additionally, assumptions about THN are often reported as negative, partially due to stigmatized assumptions of PWUO [20, 50–52]. Regulations for easier naloxone access is associated with lower opioid related death [3], which highlights the prescription-only status of THN as a main barrier. This is also evident in the low percentage of PWUO of 63,5% who got naloxone after the naloxone training. Allowance to distribute naloxone by harm reduction services and the availability of naloxone as an over-the-counter drug are crucial for the success of future THN-programs.

### Limitations

There were challenges in precisely identifying and categorizing participating organizations due to inconsistencies in naming and the difficulty of determining whether they should be considered distinct entities. Even the German Drug Services Statistics cannot definitively determine the number of drug service organizations in Germany [53]. Not all organizations participating in NALtrain documented their naloxone trainings. The extent is unknown. Furthermore, participation in the evaluation was mandatory only in Bavaria, where state funding was tied to the project. As a result, naloxone trainings conducted in Bavaria may have been documented more comprehensively compared to other states. Additionally, differences between private prescriptions and those covered by statutory health insurance were not always clearly understood, resulting in the exclusion of this topic from detailed analysis. Distributed naloxone was only counted when naloxone was directly provided after the naloxone training. This results in an underreporting of real naloxone access. It was not possible to link the documented naloxone trainings to the staff trained in the project, which further limits the analysis of the implementation process.

## Conclusion

The presented findings suggest, that low-threshold THN access and a broader involvement of physicians could be crucial to widespread THN. Regulatory authorities should prioritize enabling over-the-counter access to naloxone to remove barriers and expand availability for PWUO. For long term implementation of THN a central and ongoing coordination, support and follow-up is vital. Within this, tailored strategies for different settings can be developed and people with high risk of overdose should be prioritized. Efforts by future initiatives should focus on educating physicians, which could lead to greater involvement of medical settings. Future research should focus on the demands of different settings to implement THN. Considering the proportion of organizations offering THN, they can still be classified as “early adopters” [54]. These may serve as role models for the broader majority.

## Data Availability

The datasets used and analysed during the current study are available from the corresponding author on reasonable request.
